# FLASH/casp8ap2 Is Indispensable for Early Embryogenesis but Dispensable for Proliferation and Differentiation of ES Cells

**DOI:** 10.1371/journal.pone.0108032

**Published:** 2014-09-19

**Authors:** Yoshitaka Minamida, Masataka Someda, Shin Yonehara

**Affiliations:** Laboratory of Molecular and Cellular Biology, Graduate School of Biostudies, Kyoto University, Yoshida-Konoe, Sakyo, Kyoto, Japan; German Cancer Research Center, Germany

## Abstract

FLICE/caspase-8-associated huge protein (FLASH)/casp8ap2 is involved in various cellular functions, such as cell cycle progression, transcriptional regulation, the regulation of apoptosis, and the regulation of histone gene expression. The down-regulated expression of FLASH has been shown to inhibit cell cycle progression in the S phase in many kinds of mice and human cell lines and the inhibition of cell cycle progression may be attributed to the suppressed expression of replication-dependent histone genes. We here demonstrated that the induced knockout of FLASH never affected cell cycle progression in ES cells, in which the expression of core histone genes was decreased to levels similar to those in human KB cells sensitive to the knockdown of FLASH. In addition, the FLASH conditional knockout ES cells could differentiate normally into not only mesodermal and endodermal cells, but also trophoblasts. In order to investigate the function of FLASH in early embryogenesis *in vivo*, we also examined a FLASH mutant mouse, in which FLASH mutant allele did not express FLASH mRNA in embryos and most adult organs, except for the testis. FLASH mutant embryos died between E3.5 and E8.5. Furthermore, the *in vitro* cultivation of FLASH mutant embryos generated by *in vitro* fertilization showed embryonic lethality at the pre-implantation stage by inhibiting the hatching of embryos and their adherence to substrates. Taken together, these results indicate that FLASH plays an important role in early embryogenesis, but is not essential for either the proliferation or differentiation of ES cells.

## Introduction

FLICE/caspase-8-associated huge protein (FLASH)/casp8ap2, which was originally identified as a caspase-8-assoiated protein, is a high molecular weight protein with both a putative nuclear export signal (NES) and nuclear localization signal (NLS) [1]. FLASH is conserved in humans, mice, and chickens, and possibly also in *Drosophila* [2] and *Xenopus* [3]. Previous studies showed that FLASH was mainly localized in the nucleus, formed nuclear foci, and interact with other nuclear proteins with speckled localization, such as NPAT and ARS2 [4]–[8]. FLASH was reported to play a role in various cellular processes, including cell cycle progression especially in the S phase [4], [6], [9], processing of histone mRNA [2], regulation of apoptosis [1], [5], [7], [10], and transcriptional control [3], [11]–[15]. The down-regulated expression of FLASH by an RNAi or shRNA-expression method is known to induce cell cycle arrest in the S phase. FLASH was also identified as a prognostic marker of acute lymphoblastic leukemia [16], and the monoallelic deletion of *FLASH* was observed in 18% of T-cell lymphoblastic lymphoma patients who had a poor prognosis [17].

A previous study demonstrated that FLASH was mainly localized in the nucleus and in nuclear bodies with NPAT [4]. Although FLASH was initially reported to localize in Cajal bodies [18], recent studies showed that FLASH did not localize exclusively in Cajal bodies; a fraction of FLASH-containing nuclear bodies were associated with Cajal bodies [5]–[8], [10]. FLASH was also shown to localize in Histone Locus Bodies (HLBs) together with NPAT and Hinf-P, and to be an essential structural component of HLBs [4], [6], [9].

FLASH is known to play an essential role in the replication-dependent 3′-end processing of histone pre-mRNAs, and the down-regulated expression of FLASH by an RNAi or shRNA-expression method induced cell cycle arrest within the S phase [2], [6], [9]. When the expression of FLASH was suppressed, the expression levels of core histones, such as histone H2, H3, and H4, were also decreased [6], [9], [19], which, in turn, induces the arrest of cell cycle progression within the S phase.

FLASH was recently identified as an essential molecule in early embryogenesis, and the expression of histone H4 was down-regulated at both the mRNA and protein levels in *FLASH* mutant embryos [20]. In the present study, we generated and analyzed *FLASH* conditional knockout (KO) ES cell clones. These cells proliferated and differentiated normally into primitive cell lineages and the trophectoderm (TE) lineage, whereas *FLASH*-deficient mice showed an embryonic lethal phenotype at the pre-implantation stage, as previously reported. These results indicate that FLASH plays an important role in early embryogenesis, but is not essential in either the proliferation or differentiation of ES cells.

## Results

### FLASH was dispensable for the proliferation of ES cells

**Figure 1:**
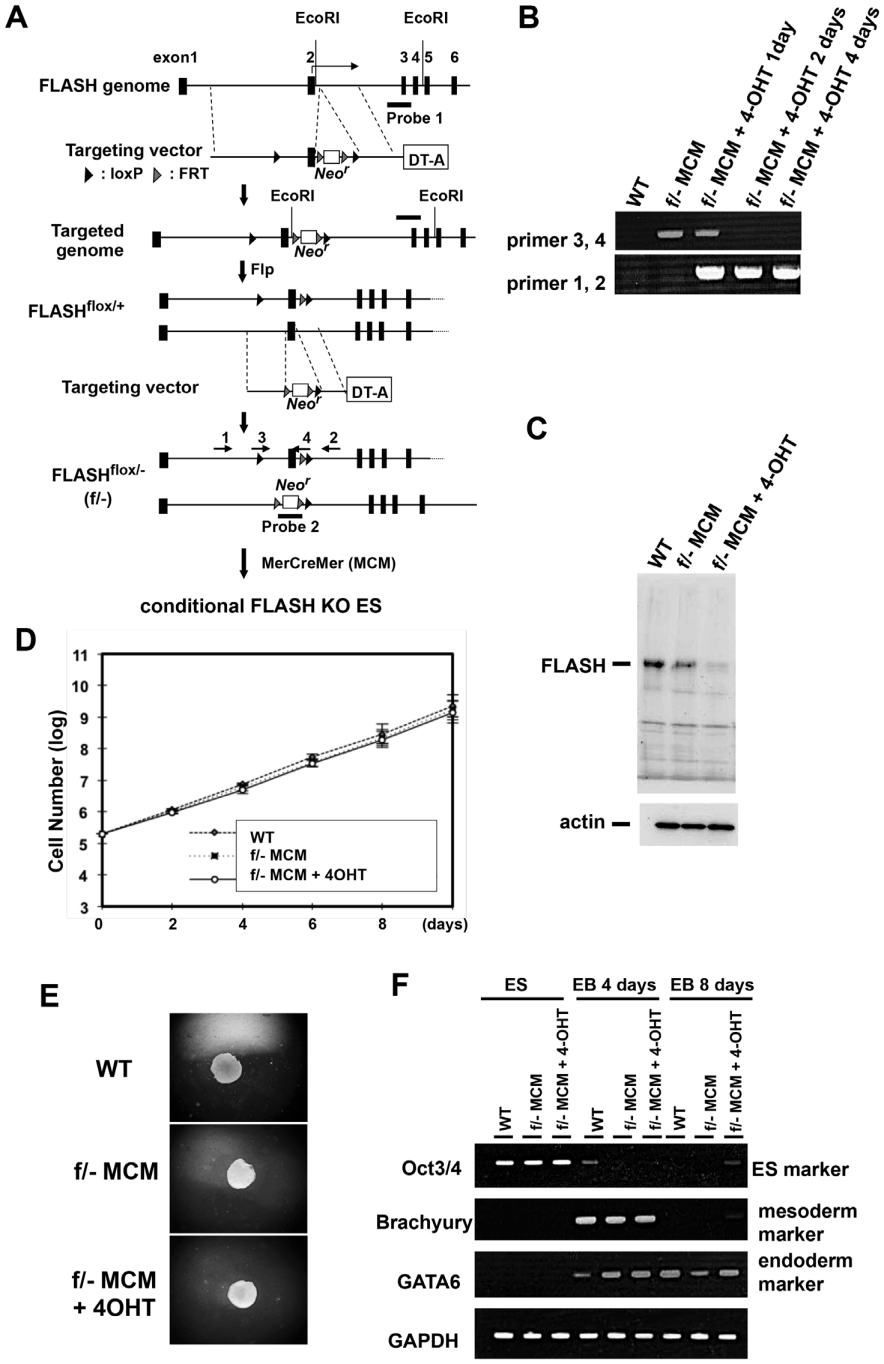
*FLASH* conditional knockout ES cells. (A) Generation of conditional FLASH knockout ES clones. FLASH^flox/-^ ES clones expressing MerCreMer were established as indicated. The activation of Cre recombinase was induced by treating cells with 4-OHT (4-hydroxytamoxifen). Arrows (number 1–4) indicate the position of the primers for genomic PCR and black boxes (Probe 1 and Probe 2) indicate the position of the probes for Southern blot analyses. *Neo^r^* and DT-A show neomycin-resistant and diphtheria toxin-A genes, respectively. (B) Genomic PCR analysis with primers 1 and 2 or primers 3 and 4 was carried out for wild-type ES (WT) and FLASH^flox/-^ (f/-) ES clones, and showed that recombination at loxP sites by MerCreMer (MCM) was correctly induced by the treatment with 4-OHT. (C) A deficiency in the FLASH protein in *FLASH* conditional KO ES cells was confirmed by Western blot analysis with an anti-FLASH monoclonal antibody. (D) Cell growth was examined after inducing the knockout of *FLASH* with the 4-OHT treatment for the indicated days. (E) Embryoid body formation was analyzed after inducing FLASH knockout with the 4-OHT treatment. Embryoid bodies were generated using the hanging drop method, and observed after a 10-day cultivation. (F) The expression of the indicated linage markers was analyzed during embryoid body formation (EB) by RT-PCR.

To examine the function of FLASH in early embryogenesis, we generated *FLASH* conditional knockout (KO) mouse ES cell clones using a gene targeting technique with the Cre-loxP system (Figure 1A). The *FLASH* gene encodes 12 exons, and exon 2 contains a translation initiation site. We initially generated *FLASH^flox/+^* ES clones in which exon 2 of *FLASH* in one allele was flanked by loxP sites. *FLASH^flox/-^* ES clones were then generated by deleting exon 2 of *FLASH* in the other allele. By using an expression vector for Mer-Cre-Mer (Mouse Estrogen Receptor-Cre-Mouse Estrogen Receptor), the treatment of *FLASH^flox/-^* ES cells with 4-OHT led to the *FLASH* gene being biallelically deleted (Figure 1B). The expression of the FLASH protein was suppressed 4 days after the treatment with 4-OHT (Figure 1C). To investigate the function of FLASH in ES cells, we examined the effects of a more than 10-day treatment with 4-OHT on the growth of *FLASH* conditional KO ES cells. In contrast to previous findings in various human and mouse cell lines [4], [6], [9], the induction of *FLASH* KO did not affect the proliferation of ES cells (Figure 1D).

### FLASH was dispensable for the differentiation of ES cells

The induced knockout of the *FLASH* gene did not affect the proliferation of ES cells. We then investigated whether *FLASH* knockout affected the differentiation of ES cells induced by Embryoid Body (EB) formation. No significant difference was observed in EB formation activity between *FLASH* KO ES cells and WT ES cells (Figure 1E). The expression levels and patterns of both an undifferentiated ES cell marker, *Oct3/4*, and differentiated ES cell markers to the mesoderm and endoderm, *Brachyury* and *GATA6*, respectively, were the same between *FLASH* WT and *FLASH* KO ES cells (Figure 1F). These results suggested that FLASH may not affect the early development of ES cells.

We then examined the differentiation activity of *FLASH* KO ES cells. *FLASH* KO ES cells could differentiate into Tuj-1-positiive neural cells, and no significant differences were observed in neuronal differentiation activity between WT and *FLASH* KO ES cells (Figure S1). We then generated beating cardiac muscle cells from WT and *FLASH* KO ES cells after the 10-day *in vitro* cultivation of EB. *FLASH* KO ES cells differentiated normally into cardiac muscle cells and no significant differences were detected between cardiac muscle cells derived from WT and *FLASH* KO ES cells (Data not shown). These results suggested that FLASH may not play an essential role in not only the proliferation, but also the differentiation of ES cells *in vitro*. In addition, FLASH may dispensable for the survival and proliferation of differentiated cells derived from ES cells.

### FLASH was dispensable for the differentiation of ES cells into trophoblasts

**Figure 2:**
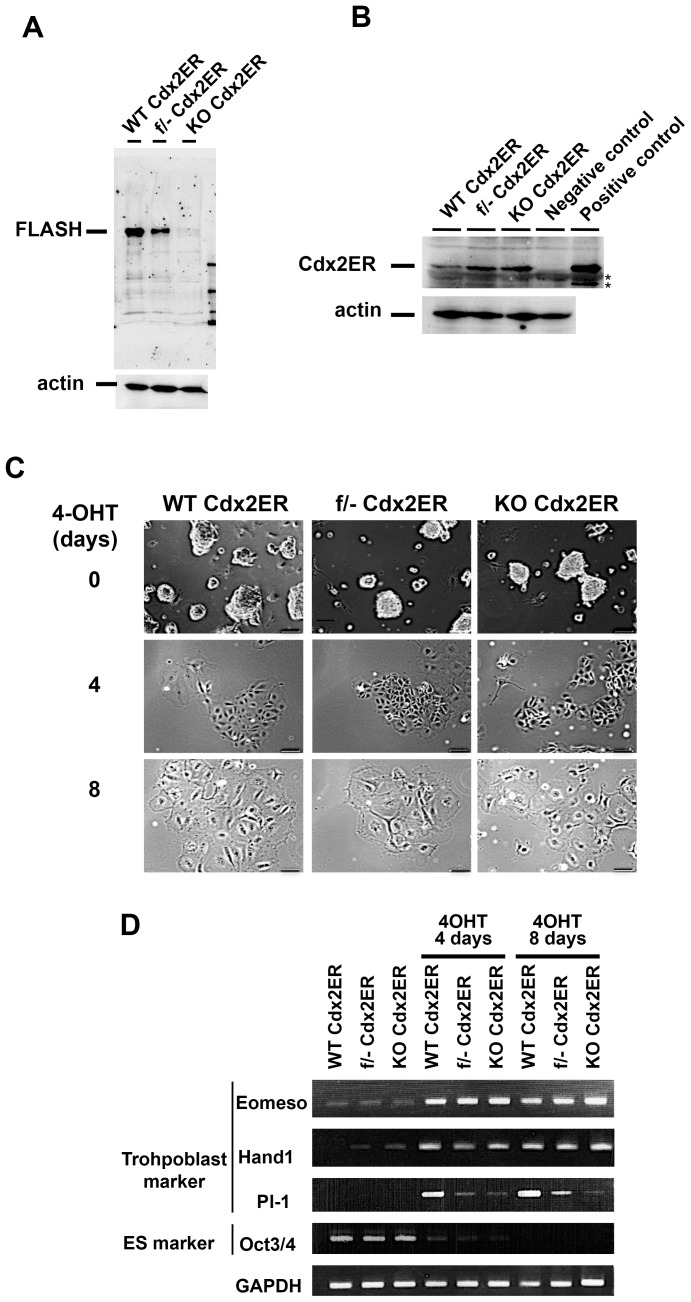
FLASH was dispensable for trophoblast differentiation of ES cells. WT, FLASH^flox/-^ (f/-), and FLASH KO (KO) ES clones, all of which could express the fusion protein between Cdx2 and the estrogen receptor (Cdx2ER) with the 4-OHT treatment, were generated. Trophoblast differentiation was induced by the treatment with 4-OHT. (A) The expression of the FLASH protein was confirmed using Western blot analysis. (B) The expression of the Cdx2ER protein was analyzed using Western blot analysis with an anti-ER antibody. The negative control showed parental WT ES cells and the positive control showed ES cells transiently overexpressing Cdx2ER. (C) Cells were cultured with LIF and 4-OHT for 4 and 8 days and examined under a phase-contrast microscopy. Scale bar, 100 µm. (D) The expression of the indicated trophoblast markers was analyzed during the induction of trophoblast differentiation by RT-PCR.

Although FLASH was dispensable for the *in vitro* proliferation and differentiation of ES cells, it was previously shown to be essential during early embryogenesis in the mouse. We speculated that the discrepancy in the phenotype between ES cells and zygotes that do not express FLASH may have been caused by the ability to form a placenta. The trophectoderm (TE) is essential for hatching from the zona pellucida and implantation, and the differentiation activity of zygotes to trophoblasts is subsequently indispensable for ontogenesis from zygotes. Cdx-2 is an essential transcription factor that regulated the formation and function of the TE [21]. The exogenous expression or activation of Cdx-2 in ES cells was previously shown to trigger differentiation into the trophectoderm lineage [22]. To investigate the role of FLASH in differentiation into trophoblasts or the survival of trophoblasts, we generated an inducible TE differentiation system in *FLASH* KO ES cell lines. A Cre recombinase-expression vector was introduced into a *FLASH ^flox/-^* (heterozygous *FLASH* KO) ES cell clone by electroporation and a single *FLASH* KO ES clone was produced. Transgenic *FLASH* wt, *FLASH^flox/-^*, and *FLASH* KO ES cells, all of which constitutively expressed the fusion protein of Cdx2 and the Estrogen Receptor (ER) (Cdx2ER), were then generated (Figure 2A, B). These clones (WT ES-Cdx2ER, FLASH KO ES-Cdx2ER and *FLASH^flox/-^* ES-Cdx2ER) could be induced to activate Cdx2 when treated with 4-OHT. After the treatment with 4-OHT, the *FLASH* KO ES-Cdx2ER cell lines were shown to similarly differentiate into trophoblasts when compared with WT ES-Cdx2ER cells (Figure 2C). The expression patterns of the trophoblast markers, Eomeso, Hand 1, and PI-1, were similar between WT ES-Cdx2ER and *FLASH* KO ES-Cdx2ER cells after the treatment with 4-OHT (Figure 2D). The induced expression level of PI-1 was significantly lower in *FLASH* KO-Cdx2ER cells than in WT ES-Cdx2ER cells, whereas Eomeso and Hand 1 levels were similar in *FLASH* KO and WT ES-Cdx2ER cells. These results suggest that *FLASH* KO ES cells can differentiate into trophoblasts following the activation of exogenous Cdx2.

### The expression of FLASH was absent in most tissues in the *FLASH* mutant mouse

**Figure 3:**
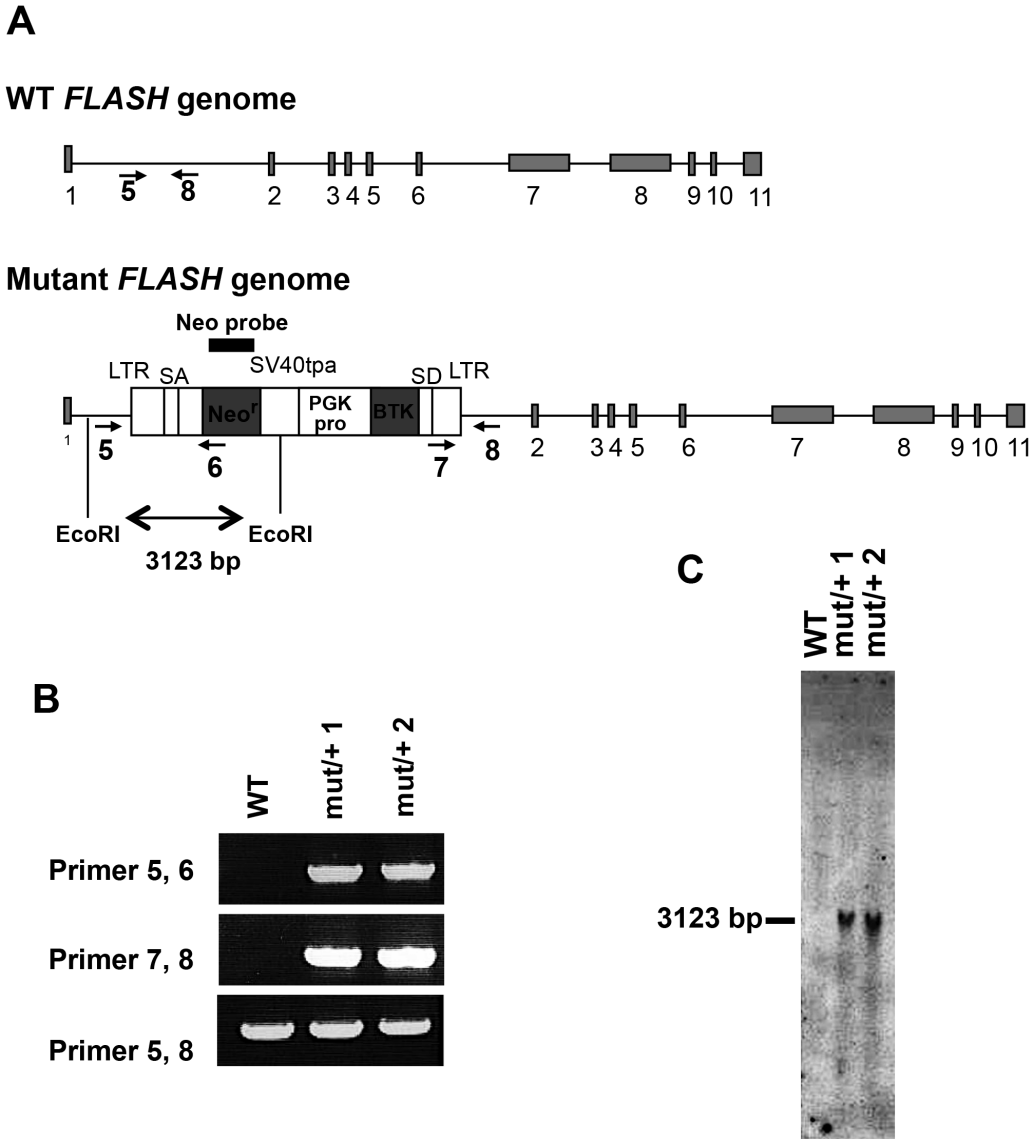
*FLASH* mutant mouse. (A) Genome structures of WT and *FLASH* mutant mice. Arrows (number 5-8) indicate the position of the primers for genomic PCR, and the black box (Neo probe) indicates the position of the probe for Southern blot analysis. LTR; viral long terminal repeat, SA: splice acceptor, SV40tpa: SV40 poly adenylation sequence, SD: splice donor. (B) Genomic PCR analysis showed that the trapping vector was inserted between exons 1 and 2 of the mouse *FLASH* gene in WT and two *FLASH^mut/+^* (mut/+1 and mut/+2) mice. (C) Southern blot analysis of WT and *FLASH^mut/+^* mice. Genomic DNA from the tail tips was digested by EcoRΙ and hybridized with the Neo probe. The 3123 bp fragment showed the trapping vector inserted into a mutant allele.

While *FLASH* mutant mice have been reported to die in the early embryonic stage [20], *FLASH* KO ES cells was shown to proliferate and differentiate normally *in vitro*. To examine the effects of FLASH during early embryogenesis, we obtained and analyzed *FLASH* mutant mouse from Lexicon Pharmaceuticals in which an OmniBank gene trapping vector was inserted in the *FLASH* allele (Figure 3A). Mouse ES cells carrying a gene trapping retroviral vector in the *FLASH* gene were found in the OmniBank, a library of gene-trapped ES cell clones identified by a corresponding OmniBank sequence tag (OST). Mice derived from the ES cell clone corresponding to OST 97730, matching the mouse *FLASH* sequence, were analyzed using inverse genomic PCR analysis and Southern blot analysis, and the results obtained showed the insertion of the gene trapping retroviral vector in intron 1 between the 789^th^ and 790^th^ bases of the mouse *FLASH* gene (Figure 3 A, B and C).

**Figure 4:**
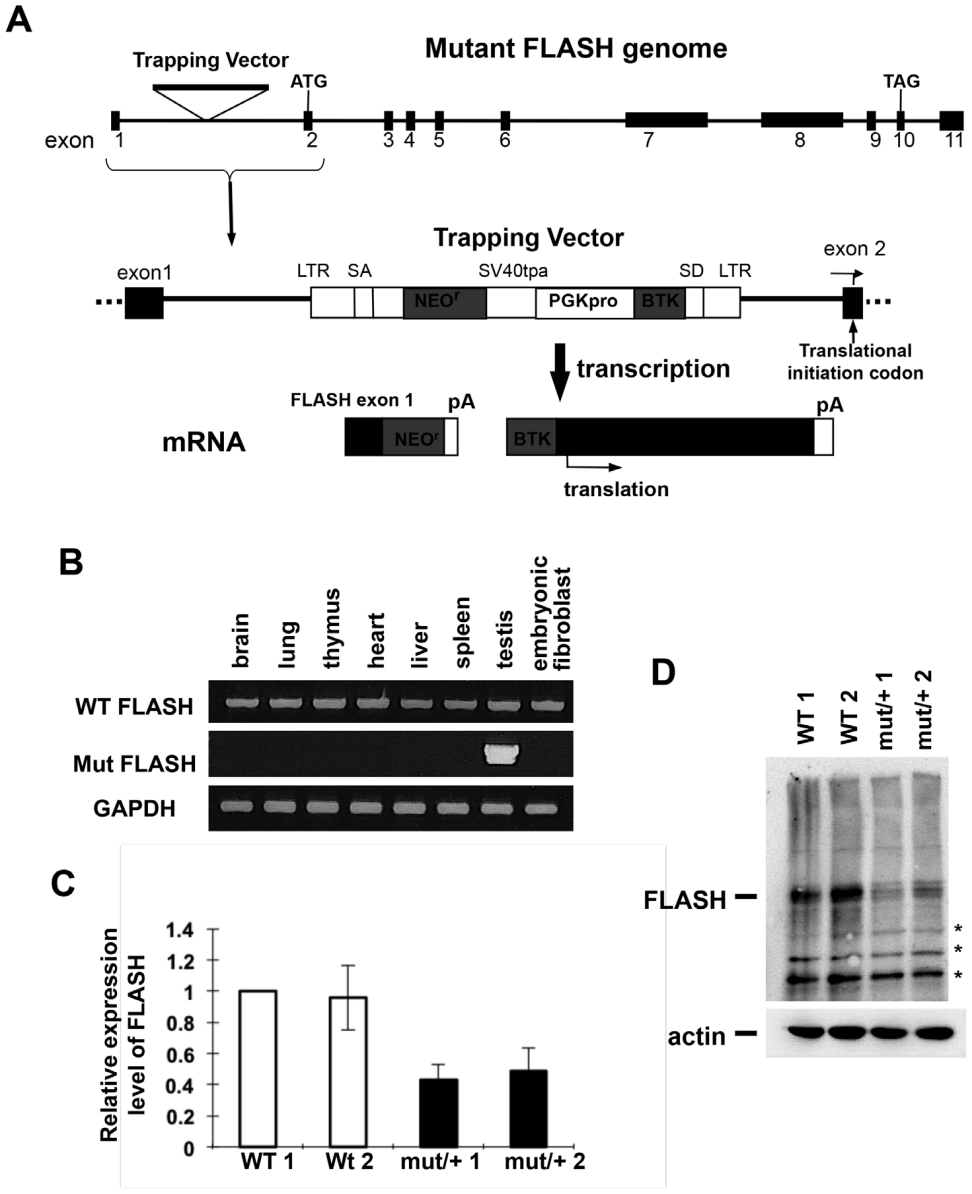
Expression of mutant FLASH in the testis only. (A) Structure of the *FLASH* mutant genome and prospective mRNAs transcribed from the *FLASH* mutant allele. (B) Total RNA was prepared from the indicated organs and embryonic fibroblasts of the *FLASH^mut/+^* mouse. WT FLASH mRNA, transcribed from the *FLASH* WT allele, and mutant FLASH mRNA were detected by RT-PCR. GAPDH was used as an internal control. (C) The amounts of FLASH mRNA in embryonic fibroblasts from two *FLASH^+/+^* mice (WT) and two *FLASH^mut/+^* mice (mut/+) were measured using qRT-PCR. (D) Western blot analysis with an anti-FLASH Ab. *, non-specific band.

The insertion of the trapping vector into the *FLASH* gene generated a fusion transcript between the Neomycin-resistant gene with the translational termination codon and exon 1 of the *FLASH* gene under the control of the *FLASH* promoter (Figure 4A). This fusion transcript only produced the Neomycin-resistant protein because exon 1 of the *FLASH* gene did not encode the translational initiation codon of *FLASH*. The trapping vector encoded a PGK promoter followed by a small piece of the *BTK* gene connected to a splice donor signal. Splicing from the donor site to the splicing acceptor site in exon 2 of the *FLASH* gene, which encoded the translational initiation codon of *FLASH*, generated fusion mRNA (mutant FLASH mRNA) containing a piece of BTK and exons 2–11 of *FLASH*, which were presumed to produce the complete FLASH protein encoded by exons 2–11 of the *FLASH* gene (Figure 4A). We then analyzed the expression of WT and mutant FLASH mRNA by RT-PCR in the various organs and embryonic fibroblasts (MEFs) of *FLASH^mut/+^* mice. WT FLASH mRNA was detected in all the organs examined and MEFs; however, mutant FLASH mRNA was only detected in the testis (Figure 4B). These results suggested that mutant FLASH mRNA was not expressed from the *FLASH* mutant allele in most tissues, except for the testis. To confirm this result, the amounts of FLASH mRNA and the FLASH protein in *FLASH^+/+^* and *FLASH^mut/+^* MEFs were quantified using qRT-PCR and Western blot analyses, respectively. The expression levels of both FLASH mRNA and protein were almost 50% lower in *FLASH^mut/+^* MEFs than in *FLASH^+/+^* MEFs (Figure 4C and D). These results demonstrated that the FLASH mutant allele did not express FLASH in most tissues, except for the testis.

### FLASH was essential for early embryonic development

*FLASH^mut/+^* mice were healthy and did not appear to be different from their WT littermates (Data not shown). Genotyping of postnatal mice after intercrossing with heterozygote mice revealed the presence of WT mice and heterozygous mice (*FLASH^mut/+^*) at the expected 1∶2 Mendelian ratio, whereas the homozygous *FLASH* mutant (*FLASH^mut/mut^*) was not detected (Table 1). The genotypes of the embryos at E8.5 (E = embryonic day) through E14.5 were analyzed by PCR after *FLASH^mut/+^* mice were intercrossed, and the results obtained revealed the absence of *FLASH^mut/mut^* embryos. On the other hand, FLASH^mut/mut^ embryos were detected at E3.5 according to Mendelian ratios. These results indicated that *FLASH^mut/mut^* embryos died between E3.5 and E8.5.

**Table 1:** Genotyping of mice derived from FLASH^mut/+^ mouse intercrossing.

		Number of genotypes in viable mice			
Age (days)	+/+	mut/+	mut/mut	ND	Total
Postnatal	29	63	0		92
E14.5	5	10	0	7	22
E13.5	5	10	0	2	17
E11.5	4	8	0	6	18
E9.5	5	11	0	4	20
E8.5	5	8	0	5	18
E3.5	11	17	7		35

The genotypes of viable mice at the indicated postnatal (P) or embryonic (E) day were determined by PCR analysis. ND; genotype could not be determined.

### *FLASH^mut/mut^* embryos at the blastocyst stage could not attach to gelatin-coated dishes or hatch from their zona pellucida

**Figure 5:**
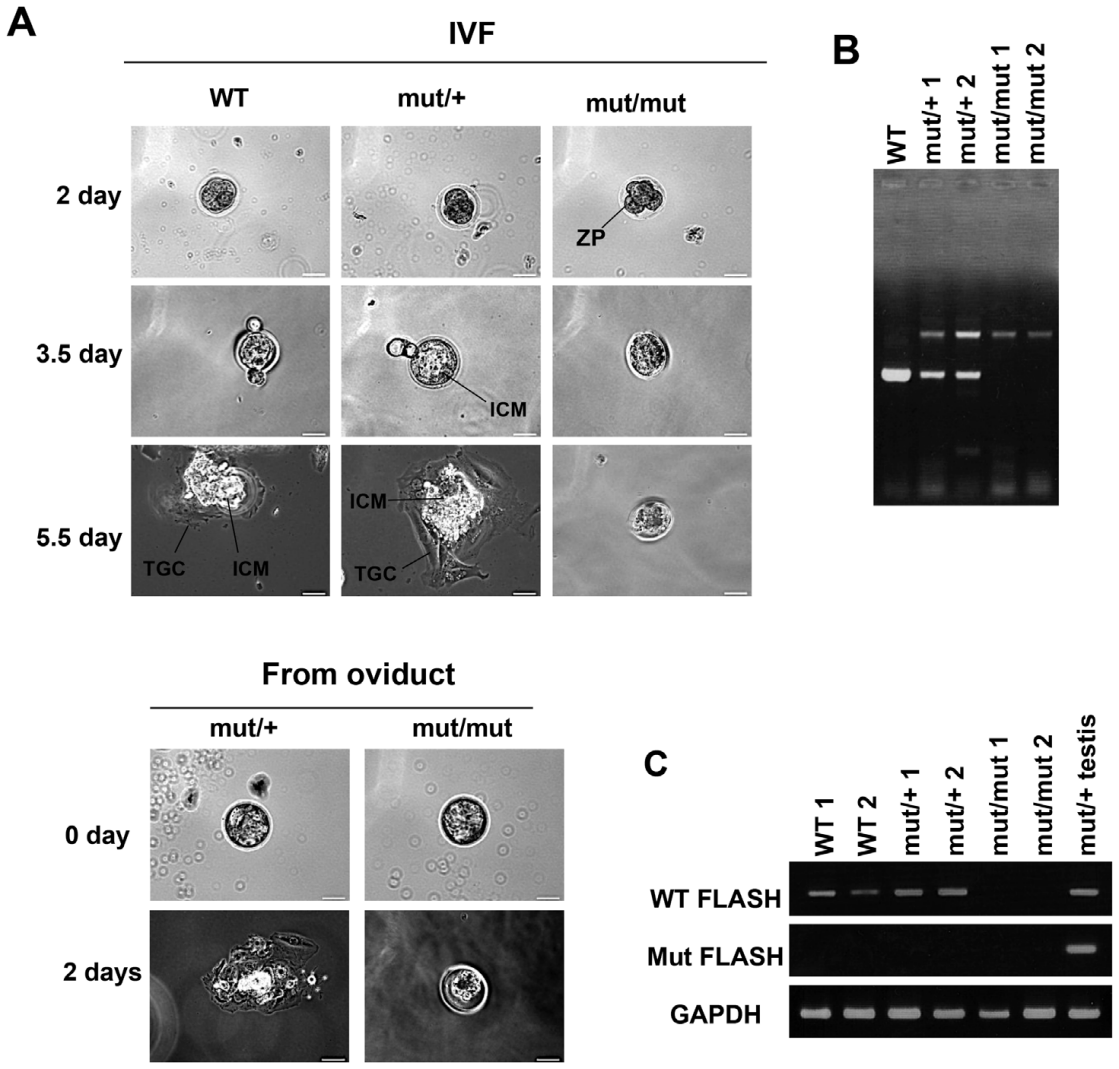
*In vitro* culture of *FLASH* mutant embryos. (A) FLASH^+/+^ (WT), FLASH^mut/+^ (mut/+), and FLASH^mut/mut^ (mut/mut) embryos 2 days after *in vitro* fertilization (IVF) or from the oviducts of FLASH^mut/+^ female mice mated 3.5 days before with FLASH^mut/+^ male mice were cultured for the indicated days in gelatin-coated dishes with ES medium, and were then observed under a phase-contrast microscope. ZP: zona pellucida, ICM: inner cell mass, and TCG: trophectoderm giant cell. Scale bar, 20 µm. (B) Genotyping of the embryos 5.5 days after IVF was carried out by PCR analysis with primers 5, 6, and 8, as indicated in Figure 3. One WT, two mut/+, and two mut/mut embryos were detected. (C) Total RNA was prepared from adult mut/+ testis, and two WT and two mut/+ embryos 5.5 days after IVF, and WT and mutant (Mut) FLASH mRNAs were detected by RT-PCR.

To further investigate the lethality of *FLASH^mut/mut^* early embryos, embryos were cultured *in vitro* in gelatin-coated dishes containing the culture medium for ES cells without leukemia inhibitory factor (LIF). These embryos were generated by the *in vitro* fertilization (IVF) of sperm and oocytes from *FLASH^mut/+^* male and female mice, respectively. Three days after fertilization, *FLASH^mut/mut^* embryos did not exhibit normal hatching from their zona pellucid and could not adhere to the culture dish, whereas *FLASH^+/+^* and *FLASH^mut/+^* embryos displayed proper hatching, appropriate adhesion to the dish, and normal progression (Figure 5A) (Table 2). The expression levels of WT and mutant FLASH mRNAs were examined in blastocyst stage embryos by RT-PCR (Figure 5B). The results obtained indicated that mutant FLASH mRNA was not expressed in *FLASH^mut/+^* or *FLASH^mut/mut^* embryos, while wild-type FLASH mRNA was expressed in *FLASH ^+/+^* and *FLASH^mut/+^* embryos. Taken together, these results clearly demonstrated that FLASH was indispensable for the pre-implantation stage by supporting the hatching of embryos and their adherence to substrates.

**Table 2:** Genotyping of embryos derived from IVF of FLASH^mut/+^ eggs and sperm.

	Number of genotypes in the embryos		
	+/+	mut/+	mut/mut
Attached	17	24	0
non-attached	4	3	14
Total	21	27	14

*In vitro* fertilized eggs were cultured for 3 days, and it was then determined whether the embryos had adhered to the culture dish.

### The transcriptional deregulation of the core histone genes was observed in *FLASH* KO ES cells

**Figure 6:**
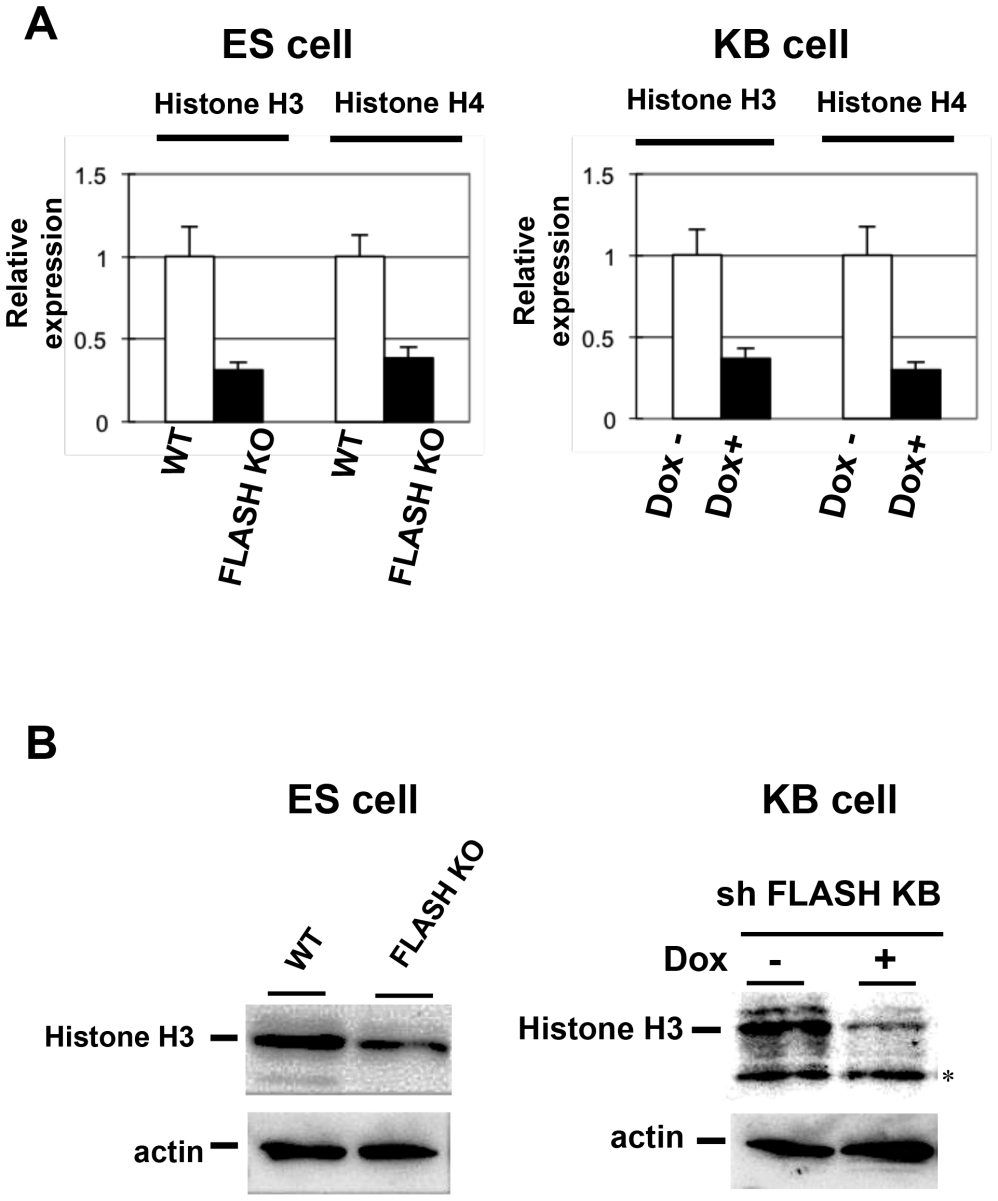
Expression of histone genes in *FLASH* KO ES and FLASH-knockdown KB cells. (A) The amounts of histone H3 and H4 mRNAs were measured by qRT-PCR in WT and *FLASH* KO mouse ES cells and human KB cells in which the expression of shRNA for FLASH was induced by the treatment with 4-OHT. (B) The amount of the histone H3 protein was measured by Western blot analysis with an anti-histone H3 polyclonal antibody. *, non-specific band.

Previous studies showed that the down-regulated expression of FLASH by RNAi and shRNA-expression methods induced cell cycle arrest in the S phase and the cell cycle arrest may be attributed to the down-regulated expression of core histone genes such as histone H3 and H4 at the mRNA level in various cell lines including human KB cells [6], [9]. In addition, the expression of both histone-H4 and H3 mRNA was shown to be lower in FLASH-aberrant embryos than in wild type embryos [20]. We examined the expression levels of histone H3 and H4 mRNA and histone H3 protein in *FLASH* KO ES cells in which cell cycle arrest was absent (Figure 6). The expression of both histone-H3 and H4 was lower in *FLASH* KO ES cells than in wild type ES cells. Consistent with previous findings [6], the induced expression of the shRNA of FLASH suppressed the expression of histone H3 and H4 genes in KB cells in which cell cycle progression was inhibited at the S phase, and the expression levels of histone H3 and H4 genes in *FLASH* KO ES cells were suppressed to a similar extent as those in KB cells expressing the shRNA of FLASH (Figure 6). These results indicated that the down-regulated expression of core histone genes was not correlated with cell cycle arrest in the S phase in FLASH down-regulated cells.

## Discussion

FLASH is known to be involved in a wide variety of physiological functions including the regulation of cell cycle progression, apoptotic signal transduction, transcriptional activation, and histone expression [1]–[15]. In the present study, we showed that FLASH was indispensable for embryogenesis at the pre-implantation stage, but was dispensable for the proliferation and differentiation of ES cells.

To investigate the function of FLASH in early embryogenesis, we generated inducible *FLASH* knockout ES cell clones. Previous studies showed that the suppression of FLASH expression by an RNAi or shRNA-expression method caused cell cycle arrest at the S phase in various cell lines [6], [9], [13]. However, our FLASH KO ES cells grew normally and cell cycle progression was normal (Figure 1D). A deficiency in the FLASH protein was examined using Western blot analysis with both an anti-FLASH monoclonal antibody and anti-FLASH polyclonal antibody (Figure 2A, data not shown). The results obtained indicated that not only the full-length FLASH protein, but also truncated forms of the FLASH protein were not generated in FLASH KO ES cells that could proliferate and differentiate normally. Previous studies demonstrated that the knockdown of FLASH caused a decrease in replication-dependent histone mRNA levels, and also that the down-regulated expression of core histones may cause cell cycle arrest at the S phase [4], [6], [9]. We then quantified the amounts of histone-H3 and H4 mRNAs and proteins using qRT-PCR and Western blot analyses, respectively, in FLASH KO ES cells (Figure 6). The suppression of FLASH expression reduced the amounts of both histone-H3 and H4 to a similar extent in not only KB cells sensitive to FLASH knockdown, but also FLASH KO ES cells. These results suggested that the down-regulated expression of S phase-specific core histone genes was not correlated with cell cycle arrest at the S phase. The molecular mechanism underlying cell cycle arrest at the S phase in FLASH-deficient cells currently remains unknown. Therefore, the mechanisms by which FLASH is involved in S phase progression and/or how ES cells with the decreased expression of core histones can proliferate normally must be clarified. We speculated that cell-cycle-independent histone variants, including histone H3.3, might be involved in restoration of normal chromatin assembly in FLASH KO ES cells. Histone H3.3 was reported to be able to substitute for S-phase specific canonical histone H3.2 in histone H3.2-deficient Drosophila, and cells in histone H3.2-deficient Drosophila could divide and differentiate when histone H3.2 was replaced by S phase-expressed histone H3.3 [23]. It is necessary to analyze the expression levels and functions of cell-cycle-independent histone variants in FLASH KO ES cells.

To investigate the physiological function of FLASH *in vivo*, we examined a FLASH mutant mouse, generated by Lexicon Pharmaceuticals, Inc., that harbored the trapping vector between exons 1 and 2 in the *FLASH* gene. We demonstrated that this mouse was not a FLASH knockout mouse, but was a mutant mouse of the FLASH promoter (Figure 4). A similar FLASH mutant mouse was used as a FLASH knockout mouse in a recent study in which FLASH was shown to be essential for mouse early development; however, the expression of FLASH mRNA and the FLASH protein was not examined [20]. In the present study, we analyzed the expression of FLASH mRNA in various organs of the mutant mouse, and the mutant allele was shown to not express FLASH mRNA in most tissues and embryos, except for the testis. Although the silencing mechanism of FLASH expression currently remains unclear, we speculated that three termination codons in the part of the BTK gene that fused with the 5′ end of FLASH mRNA may degrade mutant FLASH mRNA. This degradation of mRNA is known as nonsense-mediated decay (NMD) and was previously reported to be induced in many tissues, except for the testis [24]. The mutant FLASH mRNA was expressed in only testis conceivably through inhibition of NMD. Alternatively, we speculated that instability of nucleosomes containing a testis-specific histone variant, H3T, may be involved in the testis-specific expression of mutant FLASH mRNA, because histone H3T-containing nucleosomes were reported to be unstable [25], [26], which could enhance gene expression.

FLASH^mut/mut^ mice did not survive until birth, and an analysis of FLASH^mut/mut^ embryos revealed that they died between E3.5 and E8.5. Approximately 70% of FLASH^mut/mut^ embryos appeared to form normal blastocysts, while the other 30% showed a little lag in development. These results suggested that FLASH may be essential for mouse early development. In addition, the cultivation of embryos obtained by the *in vitro* fertilization (IVF) of FLASH^mut/+^ mice showed that FLASH^mut/mut^ embryos did not hatch from the zona pellucida or attach onto gelatin-coated dishes. In addition, approximately 30% of FLASH^mut/mut^ embryos remained at the morulae stage, whereas the remainder developed into blastocysts. In this pre-implantation stage, embryos contain maternally-produced gene products, most of which disappear at the 2–8 cell or blastocyst stage. Therefore, maternal FLASH mRNA and protein may remain and function in the blastocyst stage in FLASH^mut/mut^ embryos, and the passing of maternal FLASH may induce abnormal embryogenesis. Consistent with previous findings [20], we showed that FLASH played an essential role in embryogenesis around the pre-implantation stage. However, the mechanisms by which knockdown of FLASH expression disrupted the development of the embryo prior to implantation have not been clarified. We speculated that cell-cycle-independent histone variants, such as histone H3.3, might be involved in the mechanisms. Recently, histone H3.3 incorporation into nucleosomes was reported to be essential for parental genome reprogramming and reveal an unexpected role for ribosomal RNA transcription in the mouse zygote [27]. In FLASH KO embryo, down-regulated expression of S-phase-specific canonical histones might induce abnormal incorporation of histone H3.3 into nucleosomes and aberrantly-incorporated histone H3.3 might militate against early embryogenesis.

We previously reported that FLASH interacted with ARS2 [6] and both molecules played a role in cell cycle progression [6], [28]. In addition, Ars2 was recently reported to play a role in histone mRNA 3′ end formation and expression [29], and ARS2-deficient mice were shown to die around the pre-implantation stage, similar to FLASH-deficient mouse [30]; however, the mechanism underlying the embryonic lethality of ARS2-deficient mice remains unclear. Both FLASH and ARS2 were essential for the development of mouse embryos in the pre-implantation stage; however, it has yet to be determined whether FLASH and ARS2 have the same function in the pre-implantation stage. A detailed examination of embryos from FLASH and ARS2 knockout mice is needed in order to clarify the physiological meaning of the interaction between the FLASH and ARS2 proteins.

In order to further understand the physiological roles of FLASH, FLASH conditional knockout mice must be generated and examined, and the molecular mechanism underlying cell cycle arrest at the S phase in FLASH-deficient cells must also be clarified.

## Materials and Methods

### Ethics statement

The animal experiments were carried out in strict accordance with the protocols approved by the Animal Experiment Committee of Graduate School of Biostudies, Kyoto University (Animal Experiment Protocol No. Lif-K12009). All efforts were made to minimize animal suffering.

### Cell lines and culture conditions

MEFs [31] and KB cells [6] were cultured in Dulbecco's modified Eagle's medium (Nacalai Tesque) supplemented with 10% fetal calf serum (Sigma), 100 U/ml penicillin, and 100 µg/ml streptomycin (Nacalai Tesque) at 37°C in 5% CO_2_. A ES cell line, TT2, [32] was maintained on mitomycin C-treated MEFs in ES medium; Dulbecco's modified Eagle's medium (Gibco) supplemented with 1% fetal calf serum (JRH), 10% knockout replacement (KSR) (Gibco), 2 m M L-glutamine, 0.1 mM β-mercaptoethanol, 0.1 mM Non-essential amino acid (Gibco), 1 mM Sodium pyruvate (Gibco), and 1000 U/ml LIF (CHEMICON).

### Mice

C57BL/6 mice were purchased form CLEA Japan. *FLASH* mutant mouse was obtained from Lexicon Pharmaceuticals in which an OmniBank gene trapping vector was inserted in the *FLASH* allele. The *FLASH^mut/+^* mice on the C57BL/6 background were generated by backcrossing with C57BL/6 mice for more than 12 generations and maintained. Mice were maintained in Specific Pathogen-Free conditions.

### RT-PCR and qRT-PCR

Total mRNA for RT-PCR and qRT-PCR was prepared using ISOGEN (Nippon Gene) or the RNeasy Mini kit (Qiagen), according to the manufacturer's instructions. RT products for RT-PCR and real-time PCR were generated using the Thermo Script RT-PCR system (Invitrogen) or ReverTra Ace qPCR RT Kit (TAKARA), and a high-capacity cDNA RT kit (Applied Biosystems), respectively. RT products were analyzed with ABI PRISM7500 (Applied Biosystems). Primer sequences are shown in Figure S2. Glyceraldehyde-3-phosphate dehydrogenase (GAPDH) was used as an internal standard for all samples.

### Genotyping of mice by PCR

PCR genotyping was performed on ES cells, embryos, and tail snips that had been digested at 55°C for 4 h or overnight in lysis Buffer (10 mM Tris-HCl pH 8.0, 150 mM NaCl, 10 mM EDTA, 0.1% SDS) containing 0.4 mg proteinase K. The following primers were used for FLASH conditional KO ES cells:

primer 1 (5′-GCTATACGAAGTTATTAGGTCCCTC-3′),

primer 2 (5′-ACTCCGCGGCCCACCATTAGAATAGCTTCAACT-3′),

primer 3 (5′-AGTGGTACCTGAACTCGAGTCATCAGGCTTGCA-3′), and

primer 4 (5′-GATCCGCGGGCTACATAGTAAGACCCTGTCTC-3′).

PCR was performed using the following primers for embryos and tail snips:

primer 5 (5′-GGCGATCAGTGACAGGGGCAGTTTCC-3′) and

primer 8 (5′-AGCTTTGAAAAGGCCAGTCCCTAGCACCA-3′) to amplify a 808 bp fragment from the wild-type allele, and

primer 5 and primer 6 (5′-TGTTGTGCCCAGTCATAGCCGAAT-3′) to amplify a 1486 bp fragment from the mutant allele.

### Cultivation of isolated embryos

Heterozygous FLASH^mut/+^ female mice were superovulated using an injection of pregnant mare serum (PMS) gonadotrophin (Asuka Pharmaceuticals). After 2 days, human chorionic gonadotrophin (HCG) (Asuka pharmaceuticals) was injected, and FLASH^mut/+^ female mice were then crossed with heterozygous FLASH^mut/+^ male mice. The next day was established as embryonic day 0.5 (E0.5) if successful mating was detected by the presence of a vaginal plug. E3.5 embryos were obtained by flushing oviducts and uteri with KSOM medium (Chemicon). E3.5 embryos were placed individually onto 0.1% gelatin-coated dishes, cultured in ES cell medium without LIF, and observed under an inverted microscope (Leica).

### Cultivation of embryos obtained by *in vitro* fertilization (IVF)

PMS and HCG were injected into FLASH^mut/+^ female, and eggs were obtained for IVF by flushing the oviducts with KSOM. Sperm isolated from FLASH^mut/+^ male mice in HTF (human tubal fluid.) medium were added to the eggs. Fertilized eggs were placed individually onto 0.1% gelatin-coated dishes, cultured in ES cell medium without LIF, and observed under an inverted microscope (Leica).

### Southern blot analysis

Genome DNA of the tail snips or ES cells was digested with EcoRІ. The probe used in Southern blot analysis for the *Neo^r^* gene was generated by PCR with primers (5′-CCGGTGCCCTGAATGAACTGCAGG-3′ and 5′-CCAACGCTATGTCCTGATAGCGGT-3′). Southern blot hybridization was performed at 68°C in Rapid-Hyb Buffer (Amersham) with the ^32^P-labeled probes, followed by washing at room temperature and then at 68°C. Autoradiography was performed with FLA-7000 (Fuji Film).

### Western blot analysis

Cells were suspended in ice-cold Lysis buffer (50 mM Tris-HCl, pH 7.5 with 150 mM NaCl, 5 mM EDTA, 1% NP-40, and 0.5% sodium deoxycholate) containing a protease inhibitor cocktail (Nacalai Tesque). Cell lysates were resolved by SDS-PAGE and analyzed by Western blotting as described previously [6]. The antibodies used were an anti-FLASH monoclonal antibody (1∶1000; generated in our laboratory [6]), anti-FLASH polyclonal antibody (1∶1000; M-300; Santa Cruz), anti-actin (1∶5000; C4; Chemicon), anti-histone H3 (1∶1000; BioLegend), and anti-estrogen receptor (1∶100; HC-20; Santa Cruz). The secondary antibodies used were Alexa Fluor 488-conjugated anti-mouse or rabbit IgG (1∶5000; Invitrogen).

### Construction of the targeting vector and establishment of FLASH conditional knockout ES lines

The first and second targeting vectors described in Figure 1A were constructed using a C57Black6 BAC clone RP23-69g24 (BACPAC Resources Center). The first targeting vector contained two loxP sites at 7976^th^ bp and 12255^th^ bp of the *FLASH* gene. The targeting vector was introduced into ES cells by electroporation (0.3 KV, 75 µF; twice). After screening by G418 selection, recombinant ES clones (FLASH^flox/+^) were identified by PCR, and confirmed by Southern blot analysis. FLP recombinase encoded by an adenovirus vector was introduced into the ES clones in order to eliminate the *Neo^r^* gene. The second targeting vector containing a *Neo^r^* gene in exon 2 of the *FLASH* gene was introduced into the ES clones by electroporation, and FLASH^flox/-^ clones were identified after screening by G418 selection and PCR analysis. A pCAG-MerCreMer-IP vector encoding a fusion gene of the mouse estrogen receptor and Cre (Mer-Cre-Mer), IRES, and a *puro^r^* gene were then introduced into the FLASH^flox/-^ clones and FLASH^flox/-^ clones expressing MerCreMer were established after screening by puromycin.

### Trophoblast differentiation

A pCAG-Cdx2ER-IP vector, which was gifted by Dr. Hitoshi Niwa, RIKEN CDB, was introduced into a FLASH^flox/-^ ES cell clone, and FLASH^flox/-^ ES cells expressing Cdx2ER were identified by selection with puromycin. A pIC-Cre vector was then introduced into the ES clone by electroporation (0.25 KV, 500 µF), and a FLASH^-/-^ ES clone expressing Cdx2ER was established. The ES cells obtained were seeded at a low density (3×10^3^ cells/cm^2^) on gelatin-coated dishes without feeder MEFs, and treated with 1 µM 4-OHT for 8 days to generate differentiated trophoblast cells.

### Embryoid body formation

Embryoid bodies were generated using the standard hanging drop method as follows. ES cells were suspended in DMEM supplemented with 10% fetal calf serum, and 25 µl drops (1×10^4^ ES cells per one drop) were plated on the lid of petri dishes in regular arrays. The lid was inverted, placed over the bottom of petri dishes filled with PBS, and incubated at 37°C for 2 days. Embryoid bodies in the drops were transferred to bacterial-grade dishes and cultured.

### Neural cell differentiation

The differentiation of ES cells into neural cells was conducted using the SDIA (stromal cell-derived inducing activity) method as follows. ES cells were cultured on PA6 cells with neural differentiation-specific medium (GMEM (Gibco) containing 10% KSR (Gibco), 0.1 mM non-essential amino acids (Gibco), 1 mM sodium pyruvate (Sigma), and 0.1 mM 2-mercaptoethanol (WAKO)) for 10 days. The culture medium was exchanged every 2 days.

## Supporting Information

Figure S1 **Differentiation of FLASH KO ES cells to neural cells.** WT, FLASH^flox/-^ (f/-), and FLASH KO ES cells were differentiated into neural cells using the SDIA method. Each ES clone was seeded on OP9 cells and cultured with differentiation medium (GMEM (Gibco) containing 10% KSR (Gibco), 0.1 mM non-essential amino acids (Gibco), 1 mM sodium pyruvate (Sigma), and 0.1 mM 2-mercaptoethanol (WAKO)) for 10 days. A medium change was conducted every 2 days. Cells were stained with anti-Tuj1 Ab.
(TIFF)

Figure S2 **Primer sets for PCR.**
(TIFF)
