# Supplementary figures and images for: FLASH/casp8ap2 Is Indispensable for Early Embryogenesis but Dispensable for Proliferation and Differentiation of ES Cells
Source: PLoS One. 2014 Sep 19;9(9):e108032. doi: 10.1371/journal.pone.0108032 (PMC4169604; doi:10.1371/journal.pone.0108032)

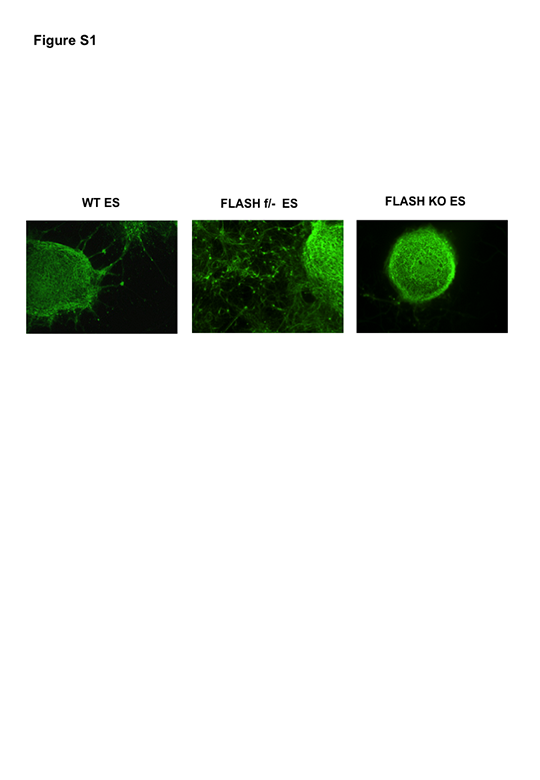

Supplement: Figure S1 — Differentiation of FLASH KO ES cells to neural cells. WT, FLASHflox/- (f/-), and FLASH KO ES cells were differentiated into neural cells using the SDIA method. Each ES clone was seeded on OP9 cells and cultured with differentiation medium (GMEM (Gibco) containing 10% KSR (Gibco), 0.1 mM non-essential amino acids (Gibco), 1 mM sodium pyruvate (Sigma), and 0.1 mM 2-mercaptoethanol (WAKO)) for 10 days. A medium change was conducted every 2 days. Cells were stained with anti-Tuj1 Ab. (TIFF) [file pone.0108032.s001.tiff]
